# 49 Post-Injury Trajectory of Body Mass Index Among Burn Survivors

**DOI:** 10.1093/jbcr/irad045.023

**Published:** 2023-05-15

**Authors:** Liam Cato, Lauren J Shepler, Lewis Kazis, Jeffrey Schneider, Oscar Suman-Vejas, Barclay Stewart, Karen Kowalske, Colleen Ryan

**Affiliations:** Boston Children's Hospital / Harvard Medical School, Brookline, Massachusetts; Spaulding Rehabilitation Hospital, Harvard Medical School, Boston, Massachusetts; Boston University School of Public Health, Rehabilitation Outcomes Center at Spaulding, Boston, Massachusetts; Spaulding Rehabilitation Hospital/Harvard Medical School/Rehabilitation Outcomes Center at Spaulding, Boston, Massachusetts; The University of Texas Medical Branch, Galveston, Texas; University of Washington, Seattle, Washington; UT Southwestern Medical Center, Dallas, Dallas, Texas; Mass Gen Hosp/Harvard Univ, Shriners Children's Boston, Boston, Massachusetts; Boston Children's Hospital / Harvard Medical School, Brookline, Massachusetts; Spaulding Rehabilitation Hospital, Harvard Medical School, Boston, Massachusetts; Boston University School of Public Health, Rehabilitation Outcomes Center at Spaulding, Boston, Massachusetts; Spaulding Rehabilitation Hospital/Harvard Medical School/Rehabilitation Outcomes Center at Spaulding, Boston, Massachusetts; The University of Texas Medical Branch, Galveston, Texas; University of Washington, Seattle, Washington; UT Southwestern Medical Center, Dallas, Dallas, Texas; Mass Gen Hosp/Harvard Univ, Shriners Children's Boston, Boston, Massachusetts; Boston Children's Hospital / Harvard Medical School, Brookline, Massachusetts; Spaulding Rehabilitation Hospital, Harvard Medical School, Boston, Massachusetts; Boston University School of Public Health, Rehabilitation Outcomes Center at Spaulding, Boston, Massachusetts; Spaulding Rehabilitation Hospital/Harvard Medical School/Rehabilitation Outcomes Center at Spaulding, Boston, Massachusetts; The University of Texas Medical Branch, Galveston, Texas; University of Washington, Seattle, Washington; UT Southwestern Medical Center, Dallas, Dallas, Texas; Mass Gen Hosp/Harvard Univ, Shriners Children's Boston, Boston, Massachusetts; Boston Children's Hospital / Harvard Medical School, Brookline, Massachusetts; Spaulding Rehabilitation Hospital, Harvard Medical School, Boston, Massachusetts; Boston University School of Public Health, Rehabilitation Outcomes Center at Spaulding, Boston, Massachusetts; Spaulding Rehabilitation Hospital/Harvard Medical School/Rehabilitation Outcomes Center at Spaulding, Boston, Massachusetts; The University of Texas Medical Branch, Galveston, Texas; University of Washington, Seattle, Washington; UT Southwestern Medical Center, Dallas, Dallas, Texas; Mass Gen Hosp/Harvard Univ, Shriners Children's Boston, Boston, Massachusetts; Boston Children's Hospital / Harvard Medical School, Brookline, Massachusetts; Spaulding Rehabilitation Hospital, Harvard Medical School, Boston, Massachusetts; Boston University School of Public Health, Rehabilitation Outcomes Center at Spaulding, Boston, Massachusetts; Spaulding Rehabilitation Hospital/Harvard Medical School/Rehabilitation Outcomes Center at Spaulding, Boston, Massachusetts; The University of Texas Medical Branch, Galveston, Texas; University of Washington, Seattle, Washington; UT Southwestern Medical Center, Dallas, Dallas, Texas; Mass Gen Hosp/Harvard Univ, Shriners Children's Boston, Boston, Massachusetts; Boston Children's Hospital / Harvard Medical School, Brookline, Massachusetts; Spaulding Rehabilitation Hospital, Harvard Medical School, Boston, Massachusetts; Boston University School of Public Health, Rehabilitation Outcomes Center at Spaulding, Boston, Massachusetts; Spaulding Rehabilitation Hospital/Harvard Medical School/Rehabilitation Outcomes Center at Spaulding, Boston, Massachusetts; The University of Texas Medical Branch, Galveston, Texas; University of Washington, Seattle, Washington; UT Southwestern Medical Center, Dallas, Dallas, Texas; Mass Gen Hosp/Harvard Univ, Shriners Children's Boston, Boston, Massachusetts; Boston Children's Hospital / Harvard Medical School, Brookline, Massachusetts; Spaulding Rehabilitation Hospital, Harvard Medical School, Boston, Massachusetts; Boston University School of Public Health, Rehabilitation Outcomes Center at Spaulding, Boston, Massachusetts; Spaulding Rehabilitation Hospital/Harvard Medical School/Rehabilitation Outcomes Center at Spaulding, Boston, Massachusetts; The University of Texas Medical Branch, Galveston, Texas; University of Washington, Seattle, Washington; UT Southwestern Medical Center, Dallas, Dallas, Texas; Mass Gen Hosp/Harvard Univ, Shriners Children's Boston, Boston, Massachusetts; Boston Children's Hospital / Harvard Medical School, Brookline, Massachusetts; Spaulding Rehabilitation Hospital, Harvard Medical School, Boston, Massachusetts; Boston University School of Public Health, Rehabilitation Outcomes Center at Spaulding, Boston, Massachusetts; Spaulding Rehabilitation Hospital/Harvard Medical School/Rehabilitation Outcomes Center at Spaulding, Boston, Massachusetts; The University of Texas Medical Branch, Galveston, Texas; University of Washington, Seattle, Washington; UT Southwestern Medical Center, Dallas, Dallas, Texas; Mass Gen Hosp/Harvard Univ, Shriners Children's Boston, Boston, Massachusetts

## Abstract

**Introduction:**

Hypermetabolism following a burn injury causes perturbations of key cardiopulmonary and cellular functions which often persist long-term. Patients are advised to increase caloric intake to address this hypermetabolic state. However, potential weight change implications from such blunt strategies are not well studied. We aimed to 1) determine factors associated with weight change from discharge and 2) understand their relationship to other physical and psychosocial measures over time. By doing so, more tailored approaches to healthy weight management could be devised.

**Methods:**

Adult participants that were enrolled in the Burn Model System (BMS) National Database after 2015 were included in the analysis. Weight was collected and BMI calculated during admission and at 0.5, 1, 2, and 5 year follow-ups. Mixed models included repeated measures controlling for correlated error of the outcomes and were used to assess the association of weight change with demographic, injury factors and psychosocial measures over time.

**Results:**

A total of 698 individuals were included in the analysis. Median age was 48 years (IQR 33, 59), 218 participants were female (31.2%), median TBSA injured was 10% (IQR 3, 25), and 388 sustained flame injury (55.6%). Median BMI was 26.75 kg/m^2^ (IQR 23.67, 31.05). During follow-up, 446 participants (64%) had a median weight gain from admission to last follow-up of 2kg (IQR -2, 6.3). There were significant differences in weight trajectory between BMS centers (p=0.0003), which was included in subsequent models. Sex, marital status, and race were not associated with weight change. Increases in burn size (p< 0.0001) and number of months working after injury (p=0.03) were associated with greater weight, while older age at time of injury (p< 0.0001) was associated weight loss. Greater weight gain was associated with higher PROMIS-29 Physical Function (p=0.002) and Neuro-QOL (p=0.03) scores (meaning better) after one year. PROMIS-29 Sleep Disturbance score was associated with weight change only between years 2-5 of follow-up (p=0.029), with higher sleep disturbance score associated with lower BMI.

**Conclusions:**

Most people living with burn injury experienced a modest weight gain after injury. Weight trajectory was impacted by several demographic factors including age, burn size, and amount time spent working, as well as physical and psychosocial measures (PROMIS-29 Physical Function, Neuro-QOL).

**Applicability of Research to Practice:**

These factors can be used to inform anticipatory guidance, diet recommendations, and exercise programs for people with burn injury. The impact of tracking weight and health goals with patients in the outpatient settings should be more closely examined.

